# Supplementary figures and images for: Manganese Overexposure Alters Neurogranin Expression and Causes Behavioral Deficits in Larval Zebrafish
Source: Int J Mol Sci. 2024 Apr 30;25(9):4933. doi: 10.3390/ijms25094933 (PMC11084468; doi:10.3390/ijms25094933)

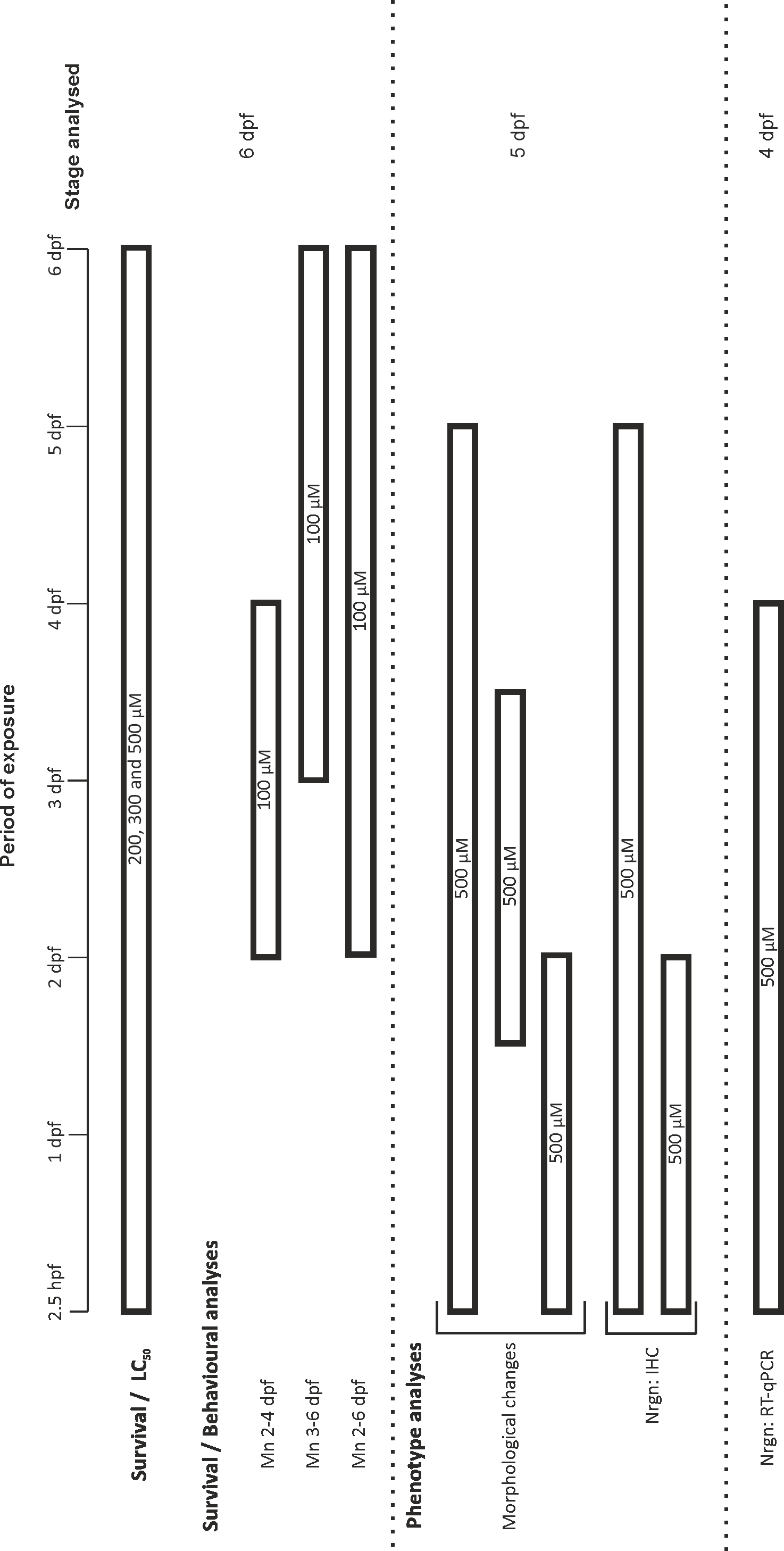

Supplement: Supplementary file 1 [file ijms-25-04933-s001.zip › Supplementary Information/Supplementary Figure 1.tif]

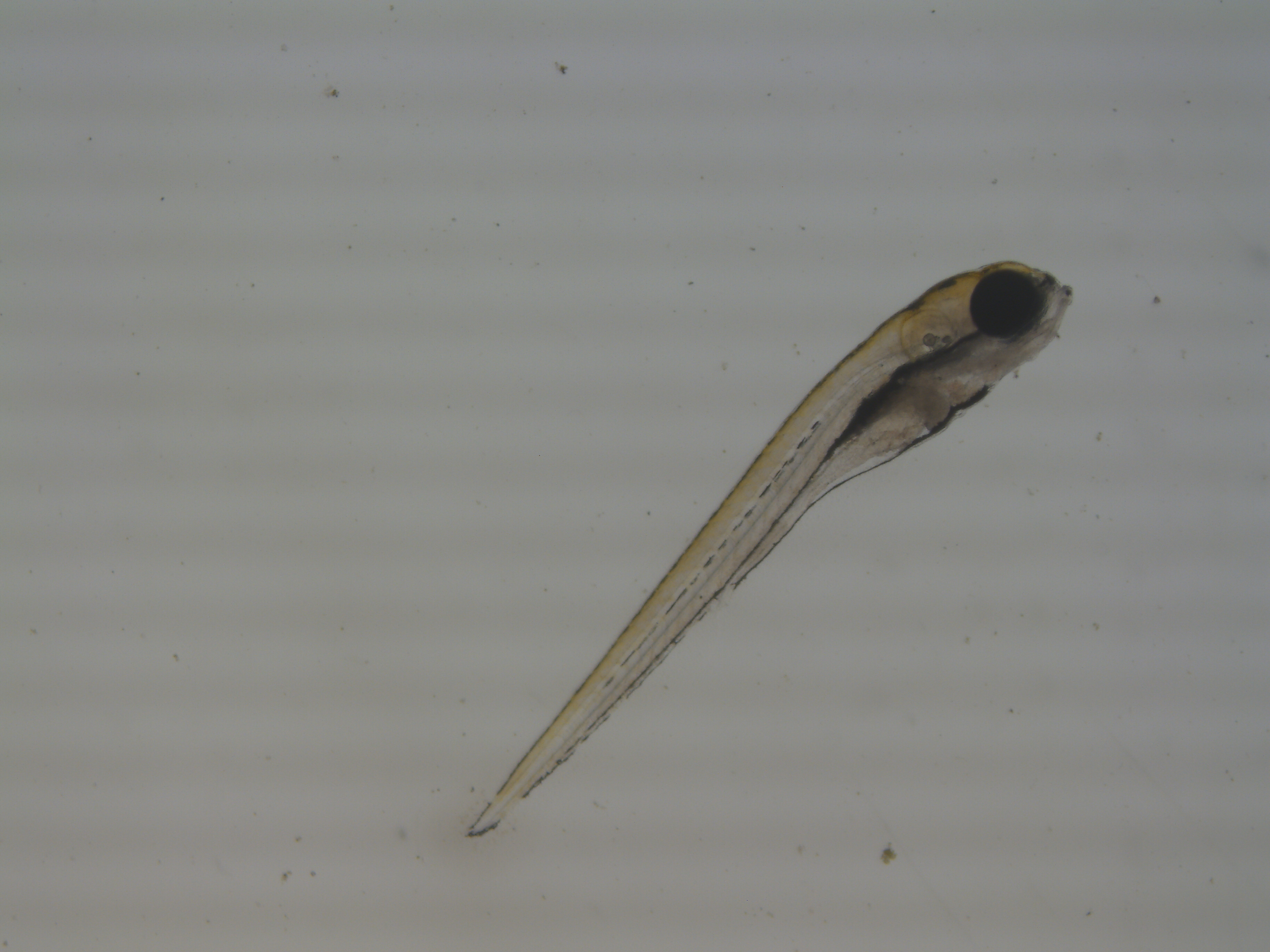

Supplement: Supplementary file 1 [file ijms-25-04933-s001.zip › Supplementary Information/Supplementary Figure 2.tif]

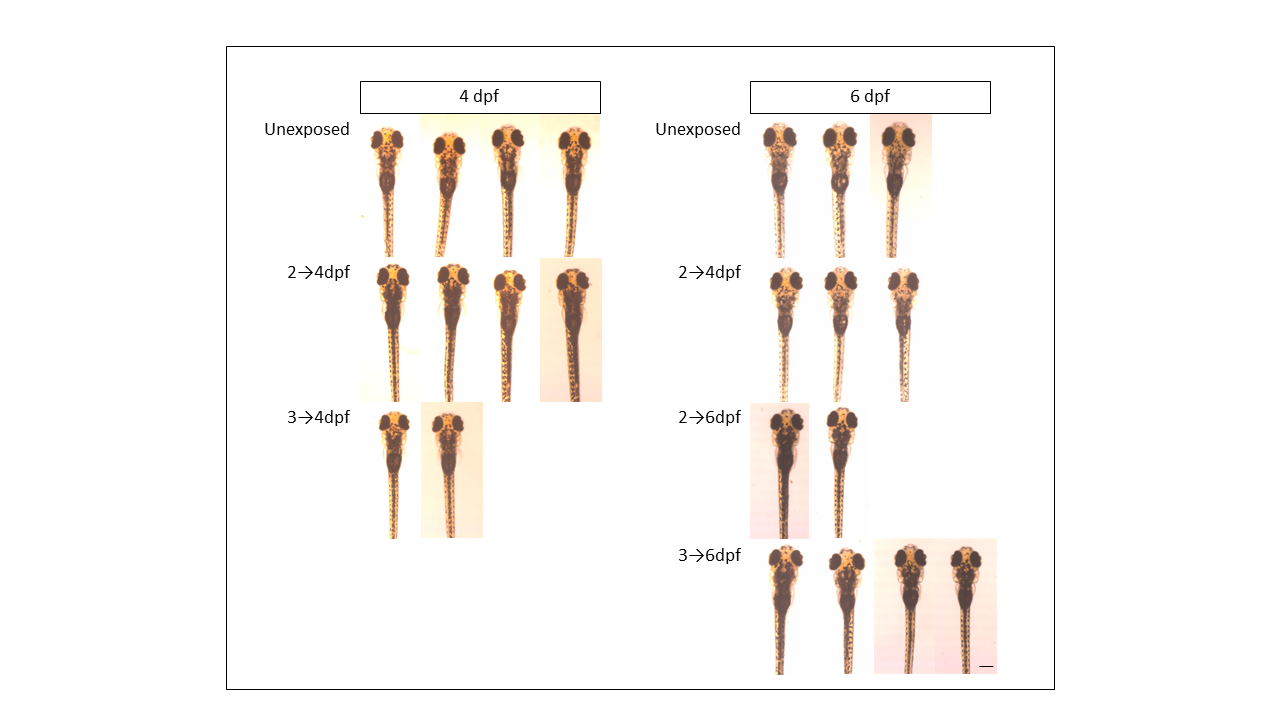

Supplement: Supplementary file 1 [file ijms-25-04933-s001.zip › Supplementary Information/Supplementary Figure 3.png]
