# Supplementary material for: Manganese Overexposure Alters Neurogranin Expression and Causes Behavioral Deficits in Larval Zebrafish
Source: Int J Mol Sci. 2024 Apr 30;25(9):4933. doi: 10.3390/ijms25094933 (PMC11084468; doi:10.3390/ijms25094933)
Supplement: Supplementary file 1 [file ijms-25-04933-s001.zip › Supplementary Information/IJMS SupplementaryInfo Alba-Gonzalez_et_al_revised_tracked.doc]

**Figure S1**

**Periods of exposure to MnCl_2_ used in this study.** *Survival analysis* at 5 dpf after exposure to increasing concentrations of MnCl_2_: 200 µM, 300 µM and 500 µM. *Behavioural analyses* at 6dpf after three different periods of exposure to MnCl_2_ (100 µM): i) from 2 dpf to 4 dpf (Mn 2-4 dpf); ii) from 3 dpf to 6 dpf (Mn 3-6 dpf); iii) from 2 dpf to 6 dpf (Mn 2-6 dpf). *Phenotype analyses* at 5dpf of embryos exposed to 500µM of MnCl_2_. Different durations of exposure were analysed: i) from 2.5 hours post fertilization (hpf) to 5 dpf; ii) from 2.5 hpf to 2 dpf; iii) from 1.5 dpf to 3.5 dpf. *Immunoreactive (IHC) fluorescence against neurogranin (Nrgn)* analysed after exposure from 2.5 hpf to 5 dpf and from 2.5 hpf to 2 dpf. *RT-qPCR* was performed by 4dpf, after exposure from 2.5 hpf to 4 dpf.;

**Figure S2**

**Characteristic appearance of zebrafish larvae at 6 dpf treated with MnCl_2_ >100µM from 2dpf.** Note absence of swim bladder inflation and larva floating on its side.

**Figure S3**

**Pigmentation pattern of MnCl_2_ exposed zebrafish larvae** at the given exposure durations at 4 and 6 dpf – additional images to show variability of pigmentation pattern with consistent hyperpigmentation upon MnCl_2_ exposure. All are dorsal views of zebrafish larvae. Scale bar: 250 µm.

**Table S1**

|  | **Survival (%)** | | | | | | | |
| --- | --- | --- | --- | --- | --- | --- | --- | --- |
|  | **Untreated larvae** | **100 µM** | | | | **200 µM** | **300 µM** | **500 µM** |
|  |  | **Mn 2-4 dpf** | **Mn 3-6 dpf** | | **Mn 2-6 dpf** |  |  |  |
| **1 dpf** | 100.00 | 100.00 | | 100.00 | 100.00 | 100.00 | 100.00 | 100.00 |
| **2 dpf** | 100.00 | 100.00 | | 100.00 | 98.85 | 100.00 | 100.00 | 100.00 |
| **3 dpf** | 100.00 | 100.00 | | 100.00 | 98.85 | 100.00 | 100.00 | 100.00 |
| **4 dpf** | 100.00 | 100.00 | | 100.00 | 98.85 | 100.00 | 100.00 | 100.00 |
| **5 dpf** | 100.00 | 100.00 | | 100.00 | 98.85 | 94.00 | 80.00 | 63.89 |

Zebrafish survival at different concentrations of MnCl_2_. Concentrations of MnCl_2_ analysed: 100 µM, 200 µM, 300 µM and 500 µM. For 100 µM MnCl_2_, three different periods of exposure were monitored: from 2 to 4dpf (Mn 2-4 dpf), from 3 to 5 dpf (Mn 3-5 dpf) and from 2 to 5dpf (Mn 2-5 dpf). For all the other concentrations tested, fish were exposed from 2.5hpf to 5dpf. n = 74 for untreated larvae. n = 64 for Mn 3-5 dpf and Mn 2-4 dpf (100 µM MnCl_2_). n = 52 for Mn 2-5 dpf (100 µM MnCl_2_). n = 50 for 200 µM and 300 µM MnCl_2_. n = 72 for 500 µM MnCl_2_.

**Table S2**

|  | **One-Way ANOVA (*P*)** | | |
| --- | --- | --- | --- |
|  | **Morphological changes after exposure to MnCl_2_**  **(500 µM)** | | |
|  | **From 2.5 hpf**  **to 5 dpf** | **From 1.5 dpf**  **to 3.5 dpf** | **From 2.5 hpf to 2 dpf** |
| **Body length (µm)** | <0.001 | 0.225 | 0.527 |
| **Olfactory organ size (normalized)** | <0.001 | <0.001 | <0.001 |
| **Eye size (normalized)** | 0.003 | 0.225 | 0.803 |
| **Brain area (µm²)** | 0.821 | - | - |
| **Telencephalic hemisphere surface (normalized)** | 0.895 | - | - |
| **Olfactory bulb size (normalized)** | 0.410 | - | - |
| **Telencephalic lobes size (normalized)** | 0.323 | - | - |

Results of the statistical analysis (p-value after One-Way Anova) for the morphological changes studied in zebrafish larvae exposed to MnCl_2_ during different periods of exposure. Normalized values were considered as the structure size to the body length ratio. n= 8 larvae per condition. (–) no information.

**Table S3**

| **Unexposed** | **Mn2→6dpf** | **Mn3→6dpf** | **Mn2→4dpf** |
| --- | --- | --- | --- |
| 0.028373333 | 25.43534333 | 22.54534333 | 5.308343333 |
| 0.025623333 | 19.31534333 | 18.82534333 | 4.577343333 |
| 0.029313333 | 23.45534333 | 24.10534333 | 5.390343333 |
| 0.044593333 | 26.90534333 | 19.88534333 | 5.159343333 |
| 0.041543333 | 20.88534333 | 15.65534333 | 3.945343333 |

**Raw data of ICP-MS analysis:** ^55^Mn concentration (ng/larva) determined at 6dpf after different exposure durations to MnCl_2_ (100µM).

**Table S4**

|  |  | |  | ***p-*value (*P*)** | | | |
| --- | --- | --- | --- | --- | --- | --- | --- |
|  |  | |  | **Untreated vs**  **Mn 2-6 dpf** | **Untreated vs**  **Mn 3-6 dpf** | **Mn 2-6 dpf vs**  **Mn 3-6 dpf** | **Untreated vs**  **Mn 2-4 dpf** |
| **Light/dark exploration** | **Number of bouts** | **Light** | | < 0.001 | < 0.001 | 1.000 | 0.438 |
|  |  | **Dark** | | < 0.001 | < 0.001 | 0.855 | 0.020 |
|  | **Mean bout velocity (mm/s)** | **Light** | | 0.980 | 0.100 | 0.203 | 0.098 |
|  |  | **Dark** | | 0.020 | 0.629 | 0.187 | 0.184 |
|  | **Bout duration (s)** | **Light** | | 0.241 | 0.890 | 0.112 | 0.607 |
|  |  | **Dark** | | 0.972 | 0.886 | 0.974 | 0.243 |
|  | **Interbout interval (s)** | **Light** | | < 0.001 | < 0.001 | 0.999 | 0.986 |
|  |  | **Dark** | | 0.02 | <0.001 | 0.952 | 0.044 |
|  | **Bout displacement (mm)** | **Light** | | 0.887 | 0.169 | 0.155 | 0.199 |
|  |  | **Dark** | | 0.388 | 0.941 | 0.665 | 0.221 |
|  | **Distance travelled (mm)** | **Light** | | < 0.001 | < 0.001 | 0.559 | 0.044 |
|  |  | **Dark** | | < 0.001 | < 0.001 | 0.825 | 0.387 |
| **OMR** | **Fraction correct angles** | **Stimulus right** | | 0.922 | 0.899 | 0.794 | - |
|  |  | **Stimulus left** | | 0.922 | 0.898 | 0.922 | - |

Results of the statistical analysis (p-value) for the behaviour paradigms studied on untreated larvae and larvae exposed to MnCl_2_ (100 μM) at during periods. At 6 dpf, statistical analyses were performed by One-Way ANOVA. Light/dark exploration showed statistical differences in the number of bouts and distance travelled (mm). Optomotor response assay (OMR) did not revealed significant differences on the fraction of correct angles neither stimuli to the right nor to the left. P < 0.05 were considered as statistically different. (-) no information;

**Table S5**

| **Primer** | **Sequence (5' → 3')** | **Amplicon size (bp)** | **Amplification efficiency (%)** |
| --- | --- | --- | --- |
| zf_nrgna_Fw | GCGAGCCGAAGTAGCTAAGA | 168 | 92.15 |
| zf_nrgna_Rv | ATGTGTGAGTGAGTCTGCCA |  |  |
| zf_nrgnb_Fw | CTCCCTGCAGAAACAGCAGA | 143 | 105.68 |
| zf_nrgnb_Rv | GGCAGAGGTGTAGGCTCAAG |  |  |
| zf_ef1α_Fw | GTACTTCTCAGGCTGACTGTG | 136 | 98.20 |
| zf_ef1α_Rv | ACGATCAGCTGTTTCACTCC |  |  |

Primers used for RT-qPCR and their amplification efficiency. Fw – Forward primer. Rv- Reverse primer. bp- base pair
